# Auxin biosynthesis by *Microbacterium testaceum* Y411 associated with orchid aerial roots and their efficacy in micropropagation

**DOI:** 10.3389/fpls.2022.1037109

**Published:** 2022-11-28

**Authors:** Archana Yadav, Kalpataru Dutta Mudoi, Niraj Kumar, Sachin Rameshrao Geed, Parishmita Gogoi, Rabin K. Sharma, Ratul Saikia

**Affiliations:** ^1^ Microbial Biotechnology Laboratory, Biological Sciences and Technology Division, Council of Scientific & Industrial Research (CSIR)-North East Institute of Science and Technology, Jorhat, Assam, India; ^2^ Department of Applied Biology, University of Science and Technology, Meghalaya, India

**Keywords:** *Microbacterium testaceum*, auxin, orchids, *Rhynchostylis retusa*, *in vitro* propagation

## Abstract

Root-associated bacteria strongly affect plant growth and development by synthesizing growth regulators and stress-relieving metabolites. The present study is mainly focused on assessing aerial root-associated bacteria of *Rhynchostylis retusa* (L.) Blume is an endemic epiphytic orchid responsible for auxin production and influencing plant growth. A bacterial isolate, *Microbacterium testaceum* Y411, was found to be the most active producer of indole-3-acetic acid (IAA). The maximum IAA production (170µg/mL) was recorded with the bacterium at optimum process parameters such as pH 7, temperature 30°C, and tryptophan 1000 µg/mL in a culture medium for 48 h. The extracted auxin was purified and analyzed by FT-IR, HPLC, and HR-MS, indicating bacterial auxin has a similar mass value to 4-chloroindole-3-acetic acid auxin. Furthermore, the bacterial auxin was tested on *in vitro* propagation of orchid, *Cymbidium aloifolium*, and 90% seed germination was recorded in Murashige and Skoog’s medium supplemented with bacterial auxin. The novel results obtained in this study are used for agricultural applications and the *Microbacterium testaceum* Y411 is a valuable biotechnological resource for a natural auxin.

## Introduction

Plants host diverse microbial communities in different plant parts, mainly roots, phyllosphere, fruits, seeds, and flowers. In such beneficial interactions, microorganisms play a valuable role because of their ability to produce plant hormones, fix atmospheric nitrogen, enhance minerals acquisition, and induce resistance to diseases. Such partners have often considered plant growth-promoting rhizobacteria (Patten and Glick, 1996). Plant hormones control the entire physiology of plants, from seed germination to fruiting response. Auxins are known to be the most effective plant growth stimulators. Indole acetic acid (IAA) is a naturally occurring auxin regulating plant development (Woodward and Bartel, 2005). It was reported that over 80% of rhizosphere bacteria are capable for producing IAA (Glick et al., 1999; Pavlova et al., 2017). Besides, bacterial auxin synthesis increases root biomass, allowing the plant to absorb water and minerals for cultivation of bacterial colonization (Spaepen and Vanderleyden, 2011). In addition, auxin enhances plant tolerance to abiotic and biotic stresses (Numan et al., 2018). Accordingly, microbial auxin has been investigated as a biological asset for agricultural use (Lee and Lee, 2010). In most of the circumstances, tryptophan monooxygenase and indole-3-acetamide hydrolase tryptophan into IAA *via* indol-3-acetamide, which is required for bacterial auxin production (Morffy and Strader, 2020).

Many bacterial strains, including pathogenic and parasitic as well as free-living microbes, are capable to produce auxin (Kunkel and Harper, 2018; Çakmakçı et al., 2020). The potential genera are *Azotobacter, Enterobacter, Alcaligenes, Agrobacterium, Pseudomonas, Bacillus, Azospirillum, Xanthomonas, Acetobacter, Rhizobium, Bradyrhizobium, Paracoccus, Arthrobacter, Herbaspirillum, Klebsiella, Methylovorus, Aminobacter, Microbacterium, Achromobacter, Flavobacterium, Rhodococcus, Acinetobacter, Corynebacterium, Micrococcus and Methylobacterium* used for the production of plant growth stimulator (auxin) are reported in earlier literatures (Barea et al., 1976; Belimov et al., 1999; Kravchenko, 2000; Ivanova et al., 2001; Spaepen and Vanderleyden, 2011; Kunkel and Harper, 2018; Çakmakçı et al., 2020).

Orchids are epiphytic monocots belonging to Orchidaceae, the most diversified flowering plant family, with 850 genera and over 25,000 species distributed worldwide, except in deserts and Polar Regions, due to rapid diversification (Fay, 2018; Rahamtulla et al., 2020). There are over 1,350 orchid species in India, with approx. 69% of them are located in North-East India, and 150 are endemic (De and Singh, 2015). They are commercially valuable for their therapeutic virtues and attractive blossoms. Orchids are also of ecological importance due to their relationship with specialized pollinators and microorganisms for seed germination (Givnish et al., 2015; Balilashaki et al., 2022). *Rhynchostylis retusa* (L.) Blume is an epiphytic orchid listed as endangered and native to tropical Asia, particularly Assam and Arunachal Pradesh. It has also been reported that certain portions have anti-mycobacterial, anti-leishmanicidal, and antibacterial properties (Tsering et al., 2017; Joshee et al., 2019). In general, epiphytic orchid aerial roots coated with silvery velamen radicum, composed of multi-layered epidermis dead cells, gaseous aid exchange, and water as well as nutrient absorption, provide mechanical support and protection from solar radiation (Pridgeon, 1986). The aerial roots have reported 30% more bacterial population than terrestrial roots (Tsavkelova et al., 2004). The functional involvement of isolated strains was demonstrated by the orchid-associated cyanobacterial community with the high nitrogen-fixing ability and heterotrophic bacterial role production of indole acetic acid (Tsavkelova et al., 2003; Tsavkelova et al., 2007). There have been no reports of such populations in *R. retusa* yet. Furthermore, on *R. retusa* velamen roots, only algal community features were investigated for specific ecological tasks such as nitrogen fixation (Deepthi and Ray, 2020).

The present work aimed to isolate and screen potential bacterial species for auxin production associated with the aerial root of *R. retusa*. The other goal was auxin extraction, purification, and characterization using analytical techniques. In order to improve the auxin biosynthesis in bacterial growth media the optimization of process parameters such as pH, temperature, substrate, and incubation periods were planned. In addition, the extracted purified auxin efficiency was evaluated for the orchid *in vitro* propagation.

## Materials and methods

### Isolation of endophytic diazotrophic bacteria

The aerial root samples were collected during the blooming period (April-May) from naturally grown epiphytic *Rhynchostylis retusa* from Jorhat (26.74°NL; 94.14°EL), Assam, India. The climate was humid tropical, with an annual mean temperature of 28.7 °C and 274 mm of rainfall (India Meteorological Department). The roots were rinsed with tap water before sterilization for 1 min with 70% ethanol (v/v) and 5 min with 0.9% sodium hypochlorite (Gangwar et al., 2014). In order to isolate the bacteria, root pieces were taken in cold, sterilized mortar pestles and crushed with saline water (0.85% of NaCl) (Li et al., 2017). An aliquot of 100 µL from each tube up to six-fold serially diluted samples were plated on Jensen’s media and incubated for 48 h at 28°C. Following incubation, morphologically different bacterial colonies were further spotted on nitrogen-fixing bacteria (NFB) media containing DL-Malic acid; 5g/L, NaOH; 3g/L, K_2_HPO_4_; 0.4g/L, FeSO_4_.7H_2_O; 0.1g/L, MnSO_4_; 0.01g/L, NaCl; 0.02g/L, CaCl_2_.2H_2_O; 0.01g/L, Na_2_MoO_4_; 0.002g/L, Agar powder; 15g/L, with pH 6.8 ± 0.1 Bromothymol blue 2 mL of 50 mg in 10 mL 70% alcohol (Dobereiner et al., 1995). The distinctive bacterial colonies were purified with repeated streaking on the nutrient agar (NA). The pure isolates were stored at -80°C in 40% glycerol stock for further study.

### Biochemical and molecular characterization of bacterial isolate

The biochemical characterization, as Catalase, Oxidase, Methyl Red, Voges–Proskauer, Carbohydrate utilization, etc., was performed using a biochemical identification test kit (HiMedia, Mumbai, India). Gram staining and morphological characterization using a laboratory microscope, characterization results were compared by Bergey’s manual (Bergey, 1994). The genomic DNA of the bacterial isolates was extracted by the BiOstic Bacteremia DNA kit (Qiagen, Germany). Amplification of the 16S rRNA gene was carried out in a Veriti Thermal Cycler (Thermo Fischer, USA) using universal forward primer 27F (5´-AGAGTTTGATCMTGGCTCAG-3´) and reverse primer 1492 R (5´-GGTTACCTTGTTACGACTT- 3´) (Dos Santos et al., 2019). The PCR products were purified using a Qiagen PCR purification kit, and sequencing was done through Agri Genome Labs India Pvt. Ltd. (Kochi, India). The resulting sequence was compared to the gene bank database using NCBI BLAST. A phylogenetic tree was constructed by MEGA 6.0 software (Tamura et al., 2013).

### Quantitative estimation of IAA

The amount of IAA produced by the bacterial isolates was determined by the colorimetric technique using Salkowski’s method (Gordon and Weber, 1951). Bacterial strains were cultured in tryptophan-containing broth (750 µg/mL) for 96 h at 28°C, followed by 10 min centrifugation at 10,000 rpm. For quantification of synthesized indole, mainly IAA, the supernatant was mixed (1:2) with Salkowski reagent (1 mL of 0.5 M FeCl_3_ with 49 mL of 35% HClO_4_) and incubated for 15 min at room temperature. After incubation, the pink color was examined at 530 nm using Biospectrometer (Eppendorf, Germany), along with various concentrations of standard of IAA (Sigma, United States).

### Parametric optimization for auxin production

Optimization experiments were carried out by cultivating the bacterial strain at different temperatures, pH, incubation times, and substrate concentrations. Further, in order to optimize the process parameters by *Microbacterium testaceum* Y411, one parameter was altered every time while keeping other parameter constant for IAA production. The temperature was optimized (26, 28, 30, 32, 34, 36, 38, and 40°C) at pH 7, substrate 750 µg/mL for 96 h incubation at 120 rpm shaking condition. Similarly, the pH was optimized at different ranges from 3 to 10 at optimized temperature, substrate 750 µg/mL, and incubation period of 96 h. Subsequently, incubation period (24, 48, 72, 96, and 120 h) and substrate L-tryptophan concentration (250, 500, 750, 1000, 1250, 1500, 1750, and 2000 µg/mL) were optimized for auxin (IAA) production at optimum temperature and pH. All the optimization experiments were performed in triplicates to avoid experimental error.

### Seed germination: Rooting assay

*Microbacterium testaceum* Y411, the most active and stable IAA producer, was tested for its ability to stimulate seed germination of mung beans (Vigna radiata). Only healthy seeds (15 numbers) were selected, and abnormally low, discolored, and damaged seeds were removed. The bacterial culture was grown in a tryptophan medium at 30 °C for 48 h. Bacterial cells were removed through centrifugation at 8000 rpm for 10 min. Clear cell-free supernatant (auxin containing) 5 mL was used for seed germination assay in a Petri dish. One-hour sterile water-soaked seeds were placed on a paper towel dampened with water at the bottom of the Petri dish. In control, only sterile media was used over the Mung seed (15 numbers). Three replicates were used for this assay. After 24-48, h seed germination will be recorded from each assay plate as given by Xu et al. (2019).

### Extraction, purification, and characterization of auxin (IAA)

Pure colony of the bacterial strain Y411 was cultivated in tryptophan broth for 48h at 30 °C and 120 rpm. The culture was centrifuged at 10,000 rpm, and the supernatant was subjected to extraction with ethyl acetate (1:2) 2 to 3 times to obtain maximum recovery (Mohite, 2013). Further, the extract was dried in a rotary evaporator (Buchi, R215, UK) before dissolved in the optimum amount of methanol.

A silica gel G 60 F 254 pre-coated aluminum plate (Merck, Germany) was used for the solvent-extracted auxin’s in thin-layer chromatography (TLC). The dissolved methanol sample was detected on a silica plate beside an IAA standard of 2mg/mL. After 30 min in the solvent isopropanol-water (60:40 v/v), the plate was air-dried and treated with Salkowski’s reagent. The pink color spot was observed, and the *R_f_* value was calculated about the standard.

The spotted auxin was scrapped from the TLC plate and eluted with a 1:2 v/v mixture of *n*-hexane and ethyl acetate (Extra pure, EMPLURA) by SPE (solid phase extraction) using a glass column (1.0 x 12cm) packed with silica gel of 60-120 mesh as a stationary phase. Before adding the sample, the column was pre-washed with *n*-hexane. The eluted product was thoroughly dried and analyzed by HPLC, FT-IR, and HRMS.

The FT-IR (Perkin Elmer, Spectrum 100 UK) analysis of the purified sample has been carried out using transmission mode with a wavenumber ranging from 400 to 4000 cm^-1^. HPLC (Waters, USA) analysis was carried out using a C18 column (5μm×4.6×150mm). The absorbance of IAA was measured at 280 nm using a mobile phase of water and acetonitrile (40:60 v/v ratio) for 30 min at a flow rate of 1mL/min. A 20 μL sample was injected, and the retention time of the compound was compared to the IAA standard (Sigma) (Aswathy et al., 2013).

The UHPLC-HRMS (Xevo XS QT, Waters, USA) instrument (mass range 20-4000 amu) was coupled with Xevo XS QT mass spectrometer through the ESI interface. The program was used as ESI voltage 4.5 kV; capillary voltage 10V; tube lens 50V N_2_ gas was used as an auxiliary, sweep, sheath at 5,2,10 arbitrary units, respectively. The MS ions spectra were acquired using Xevo XS QT mass spectrometer in the scan range of 50 to 300 m/z. For HRMS analysis, 20 µL of control IAA and purified bacterial extracted sample were used to detect the MS spectra of IAA and its metabolites.

### *In vitro* study of bacterial auxin (IAA) on orchid tissue culture

The surface-sterilized capsule containing seeds of *Cymbidium aloifolium* (epiphytic orchid) was slit in half longitudinally (Arditti, 2008). The exposed seeds were inoculated on MS medium that contained 0.8% agar and was supplemented with a range of bacterial auxin and synthetic auxin concentrations, including 0.5, 1, 1.5, and 2.0 mg/L, respectively. BAP (1 mg/L) was used as a cytokinin by Murashige and Skoog (1962), while NAA was employed as a positive control. The flasks were incubated for 16 h under photoperiod conditions (white fluorescent light) at 25 ± 1°C in an experimental tissue culture chamber. The physical observations such as seed germination, callus formation, root length, and shoot length were investigated regularly every week for 120 days. The % germination was calculated using the following formula:


$$\%\;Germination\;=\frac{X-Y}{Y}\times 100$$

Where, X represents the number of seeds inoculated

Y represents the number of seeds germinated

### Statistical analysis

One-way ANOVA was conducted, followed by Tukey’s post-test for each treatment using the SPSS software (ver. 10.1, SPSS Inc., www.spss.com). The significance level was *p*=0.05 for the effect of culture media on various parameters such as seed germination, callus formation, root number, root length, shoot length, and survival duration.

## Results

### Indole acetic acid production

**Figure 1:**
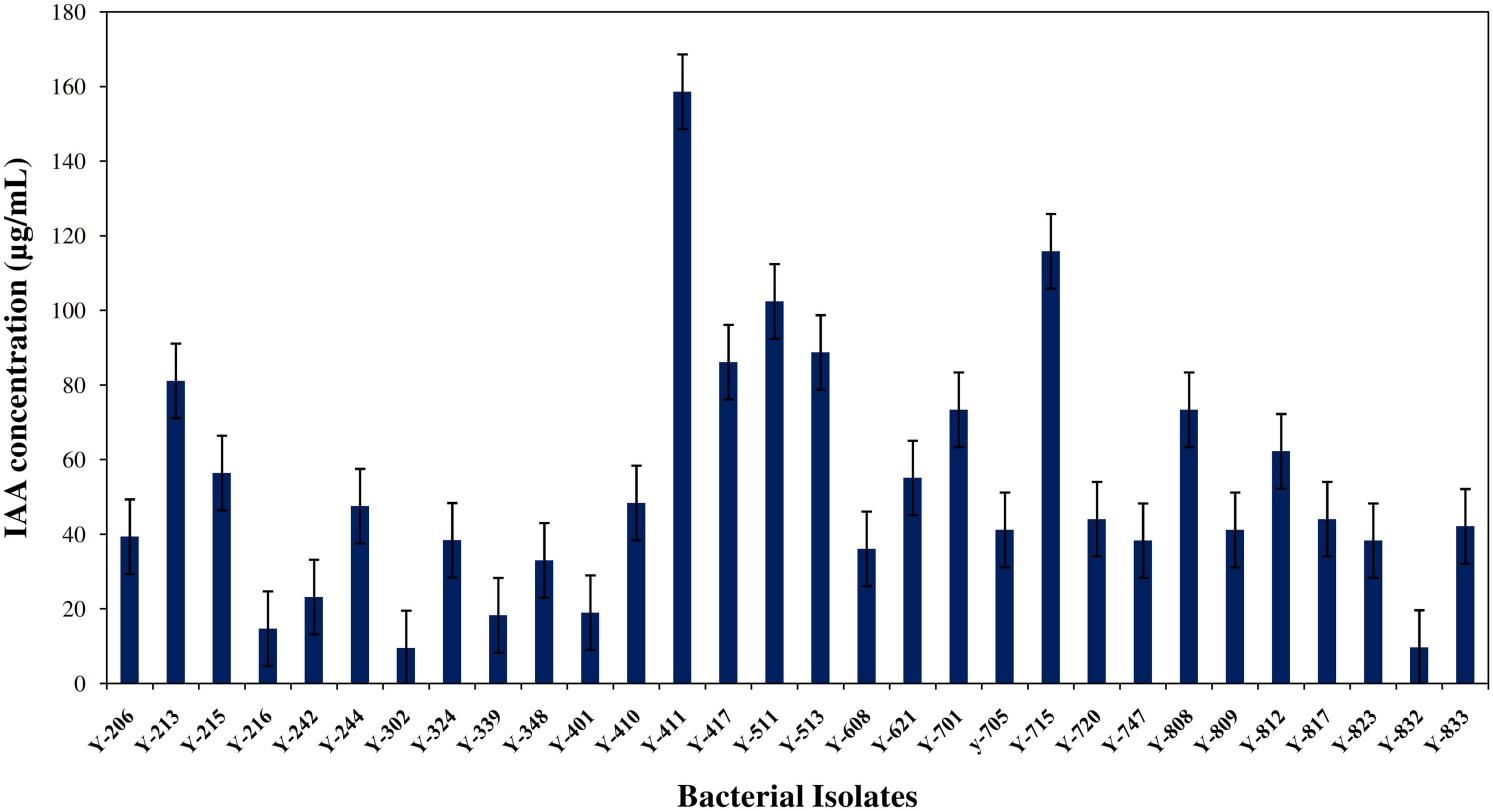
Quantitative estimation of bacterial auxin (IAA) produced by isolated strains from orchid roots of *R. retusa* using tryptophan broth medium (n=3).

All the bacterial isolates were tested for IAA synthesis; the potential bacterial species were shown prolific growth and IAA production in the presence of Tryptophan broth growth media at 28°C for 96 h of incubation. The production range of IAA was between 9 to 158.62 µg/mL, and Y411 produced the highest IAA (158.62 µg/mL), followed by *Bacillus aryabhattai* Y-715 (115.82 µg/mL) the details are given in **Figure 1**.

### Identification of auxin-producing diazotrophic bacteria

**Figure 2:**
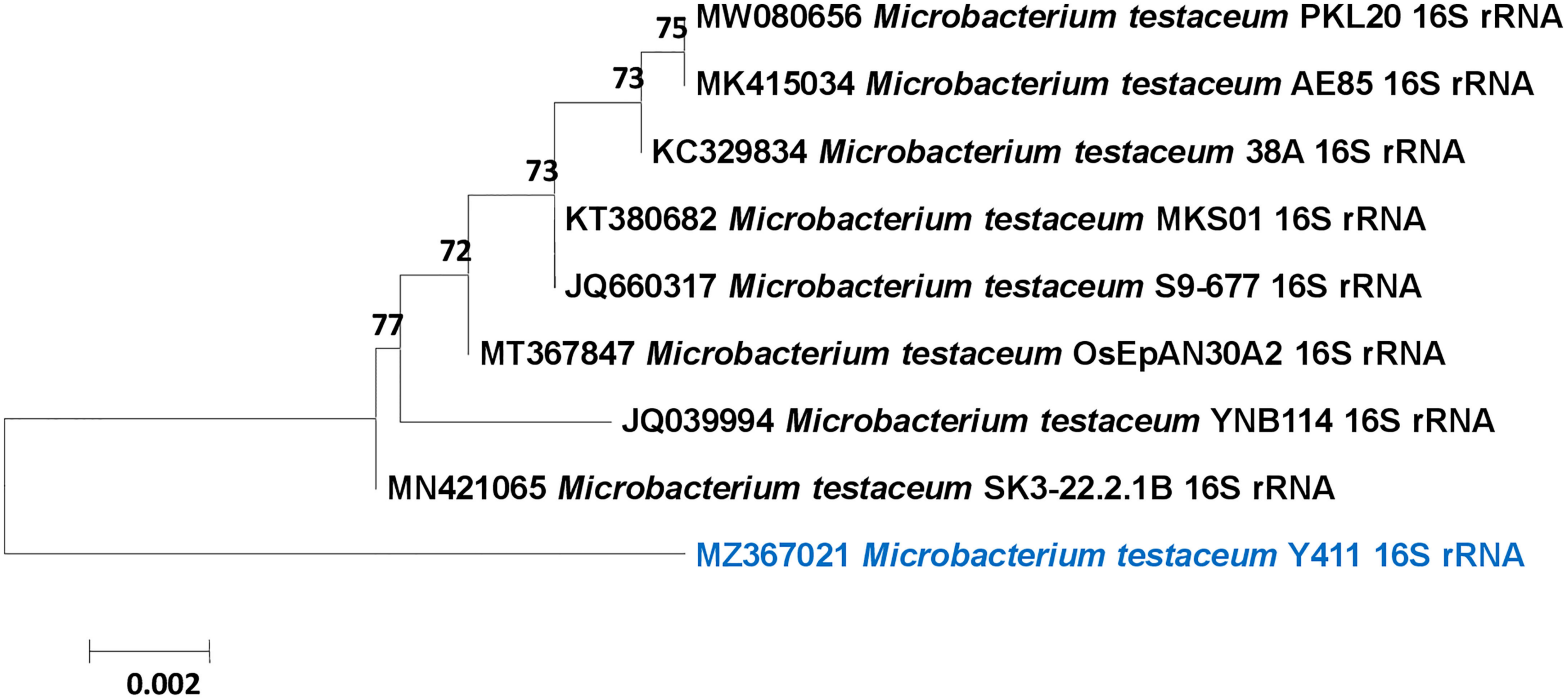
Phylogenetic tree of *Microbacterium testaceum* Y411 constructed using Neighbor-joining method showing the relationships among the 16S rRNA gene sequence of NCBI database.

The genera of isolated bacterial species are identified as *Microbacterium, Pseudomonas, Achromobacter, Brevibacterium, Arthrobacter, Stenotrophomonas, Staphylococcus,* and *Bacillus*. A highly efficient auxin-producing bacterial isolate, *Microbacterium testaceum* Y411, was identified based on morphological, biochemical, and molecular characterization. The isolate was morphologically rod-shaped and non-motile. The biochemical characterization revealed that the isolate was yellowish-orange pigmented, gram-positive with catalase-positive, and oxidase-negative. The strain can use glucose, sucrose, arabinose, mannitol, and rhamnose and grow in malic acid media **(** **Table 1** **).** The 16S rRNA gene sequence (1459 bp) was compared with the NCBI database, and a phylogenetic tree was constructed with a total branch length of 10.92302698 using the neighbor-joining method to see them closely related to homologous sequences of GenBank **(** **Figure 2** **)** (Saitou and Nei, 1987). The strain was identified as *Microbacterium testaceum* from NCBI data base (Pisarska and Pietr, 2015). A phylogenetic tree was constructed with a bootstrap value of 1000 replicates shown next to the branches. The resulting dataset had 1400 locations of 10 nucleotide sequences, with codon positions 1^st^+2^nd^+3^rd^+Noncoding included (Tamura et al., 2013). The sequence was deposited to NCBI GenBank with accession number MZ367021.

**Table 1:** Biochemical characterization of *Microbacterium testaceum* Y411.

Characteristics	Activity
Gram test	+
Catalase	+
Oxidase	–
Indole	+
Methyl Red	+
Voges–Proskauer	–
Citrate	–
Nitrate reductase	+
**Carbohydrate utilization**	
Glucose	+
Adonitol	–
Arabinose	+
Lactose	–
Sorbitol	+
Mannitol	+
Rhabinose	+
Sucrose	+
IAA production	+
Nitrogen Fixation	+
Phosphate solubilization	+
Siderophore production	+
HCN Production	+

### Seed germination and root assay

**Figure 3:**
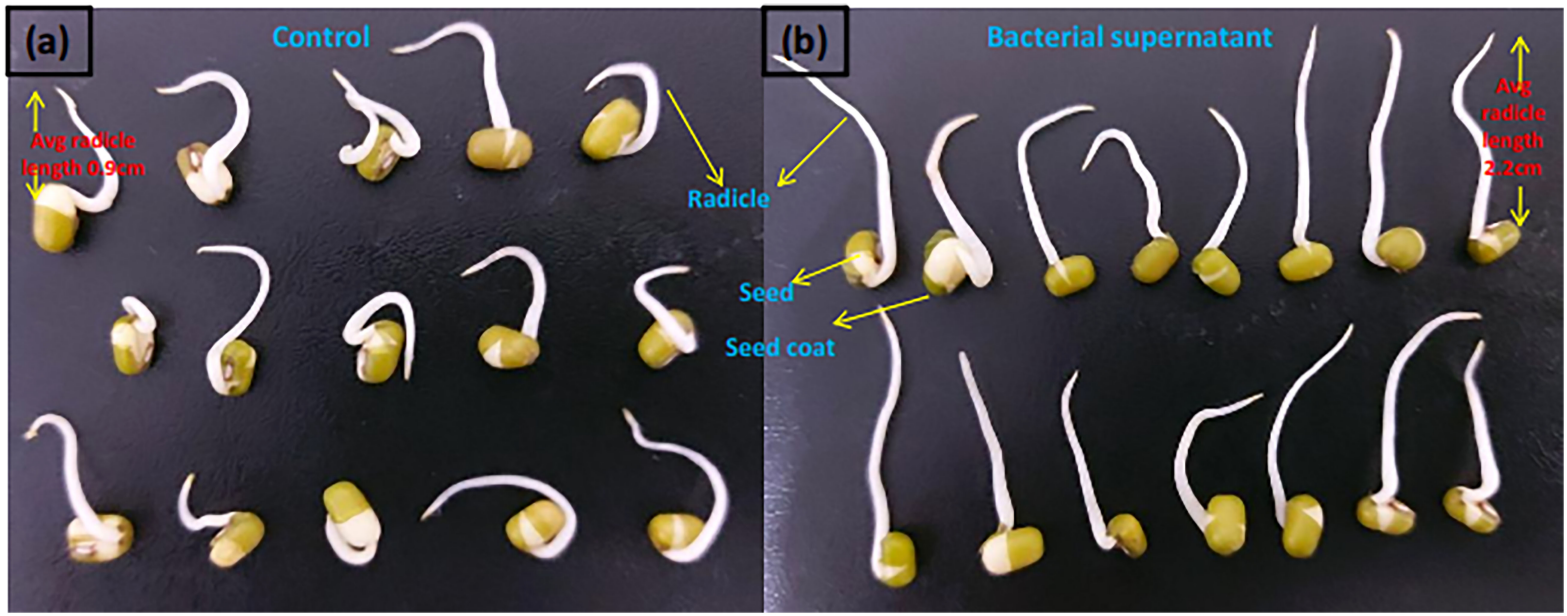
Mung (*Vigna radiata*) seed germination effect in **(A)** control and **(B)** bacterial supernatant medium.

Mung seeds and rooting assays was performed to investigate the effects of *Microbacterium testaceum* Y411 supernant (containing bacterial auxin) supported 100% seed germination with healthy radicle with an average length of 2.2 ± 0.13 cm. By contrast, in the control plate, seed germination with bent radicle with an average length of 0.9 ± 0.15 cm (see **Figure 3**).

### Optimization of process parameters

**Figure 4:**
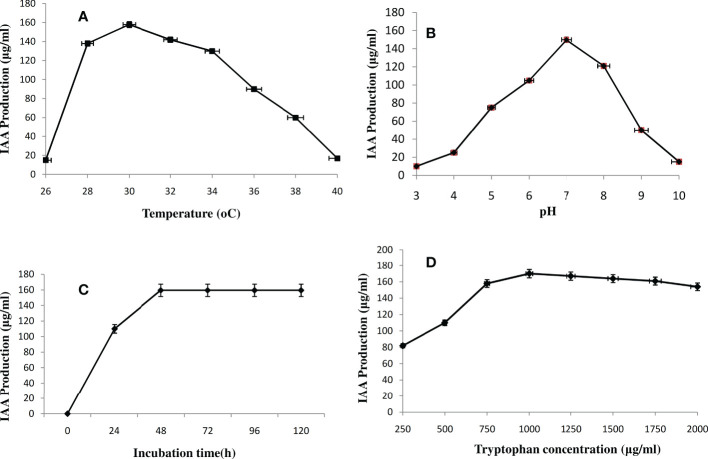
Effect of parameters **(A)** temperature (26-40°C), **(B)** pH (3-10), **(C)** incubation time (0-120h), and **(D)** Tryptophan (substrate) (250-2000μg/mL) on IAA production by *Microbacterium testaceum* Y411, values are the means (n=3) ± standard deviation.

The effect of temperature on IAA synthesis has been investigated with a range of 26 to 40°C. As the temperature was raised from 26 to 30 °C, the IAA production increased from 15 to 158 µg/mL; however, as the temperature was increased further, as shown in **Figure 4A**, the IAA production dropped from 158 to 17 µg/mL. The production of IAA was maximum at 30 °C when it produced 158 µg/mL. As pH increased from 3 to 7, IAA production increased from 10 to 150 µg/mL, decreasing as pH rose again from 7 onward (**Figure 4B**). Further, the influence of the incubation period from 0 to 120 h with 24 h intervals on IAA biosynthesis was investigated. At 48 h of incubation, IAA biosynthesis was reached 159.5 µg/mL, and at 72 h of incubation, no change in the IAA production was observed (**Figure 4C**). In the case of tryptophan concentration optimization, 1000 µg/L was the optimum concentration for IAA production by *Microbacterium testaceum* Y411 (**Figure 4D**). Finally, the maximum IAA production (170 µg/mL) was recorded at optimum temperature (30°C), pH (7.0), incubation time (48h), and tryptophan concentration (1000 µg/L) at 150 rpm by *Microbacterium testaceum* Y411.

### Partial purification and characterization of IAA

**Figure 5:**
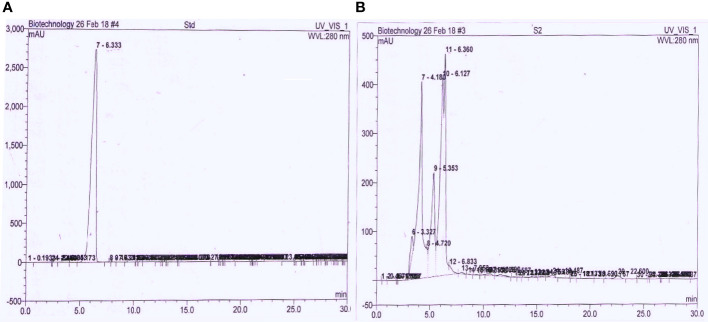
Comparative HPLC chromatograph of **(A)** control IAA and **(B)** purified extracted bacterial auxin using mobile phase water: acetonitrile (60:40) C18 column with flow rate of 1 mL/min and detected under UV at 280 nm.

TLC analysis was confirmed by observing the collected fraction’s pink spot after spraying with the Salkowski reagent. It was found that solvent-extracted bacterial auxin and standard IAA chromatographs had the same *R_f_* value of ~0.53. For maximum recovery, the bio-extract was purified in a silica gel glass column using solvent (*n*-hexane: ethyl acetate ratio 20:80). The TLC purified fraction was further analyzed through HPLC and compared with standard IAA. It was observed that purified bacterial IAA had a strong peak with a retention time of 6.36 min, which was similar to the retention time of 6.33 min of standard IAA (**Figures 5A, B**).

**Figure 6:**
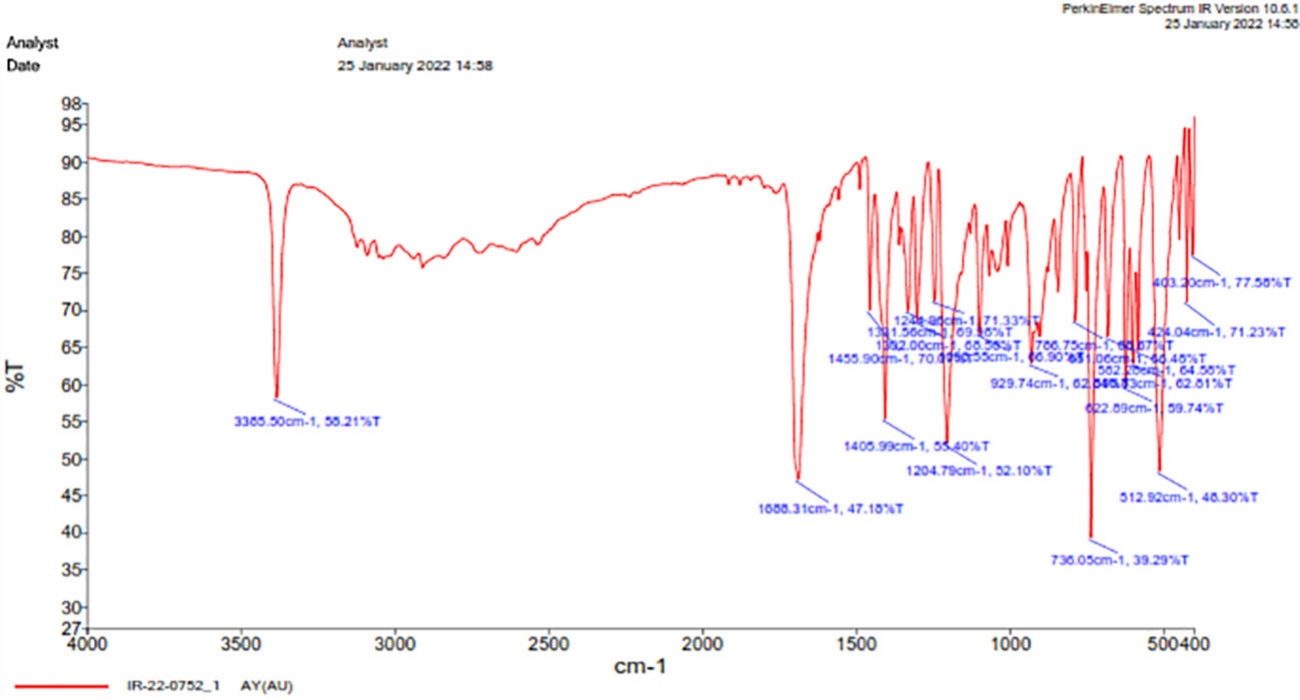
Functional analysis using FT-IR of IAA produced by *Microbacterium testaceum* Y411.

The FT-IR spectra of purified bacterial auxins were analyzed. The broad absorption band at 3385.50 cm^-1^ corresponds to the carboxylic -OH group, -C=O stretching matches the peak at 1688.31 cm^-1^ (generally obtained at 1670 cm^-1^), and -NH bends at 1455.90 cm^-1^. Furthermore, the peaks at 1302-1405 cm^-1^ were associated with the aromatic ring’s -C-H vibration, while the wave number at 1098.55 cm^-1^ was indicated for –C-H bending. The band represents -CH_2_ at 736.05 cm^-1^ (**Figure 6**).

**Figure 7:**
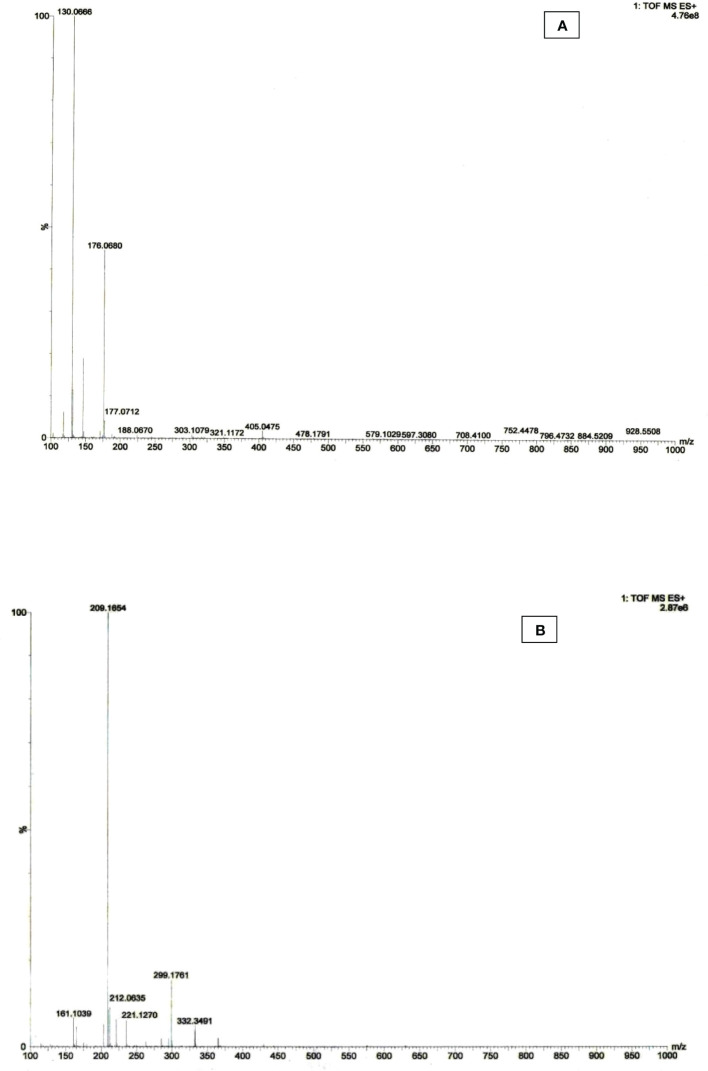
HRMS spectra of **(A)** control IAA and **(B)** extracted IAA sample of *Microbacterium testaceum* Y411.

High-resolution mass spectrometric (HR-MS) techniques using time-of-flight mass spectrum (TOF- MS ES+) provide high selectivity, mass accuracy, and structural information, allowing forging high selectivity. The target compounds were identified to confirm the identification of auxin metabolites. HRMS was used to detect the MS spectra of IAA and its metabolites in purified bacterial extracted materials. The mass-to-charge ratio of control IAA was found at *m/z* 176.06 (**Figure 7A**). The chlorinated derivative of the IAA (4-Chloro indole acetic acid) compound is composed of fragmented ions C_10_H_8_ClNO_2_ and C_10_H_9_ClNO_2_ were found at *m/z* 209.16 and 212.06, indicating notable auxin activity (**Figure 7B**). The *m/z* ratios of IAA in the bacterial sample match those of commercially available synthetic chemicals, indicating that metabolites exist.

### *In vitro* propagation of orchid using bacterial auxin

**Figure 8:**
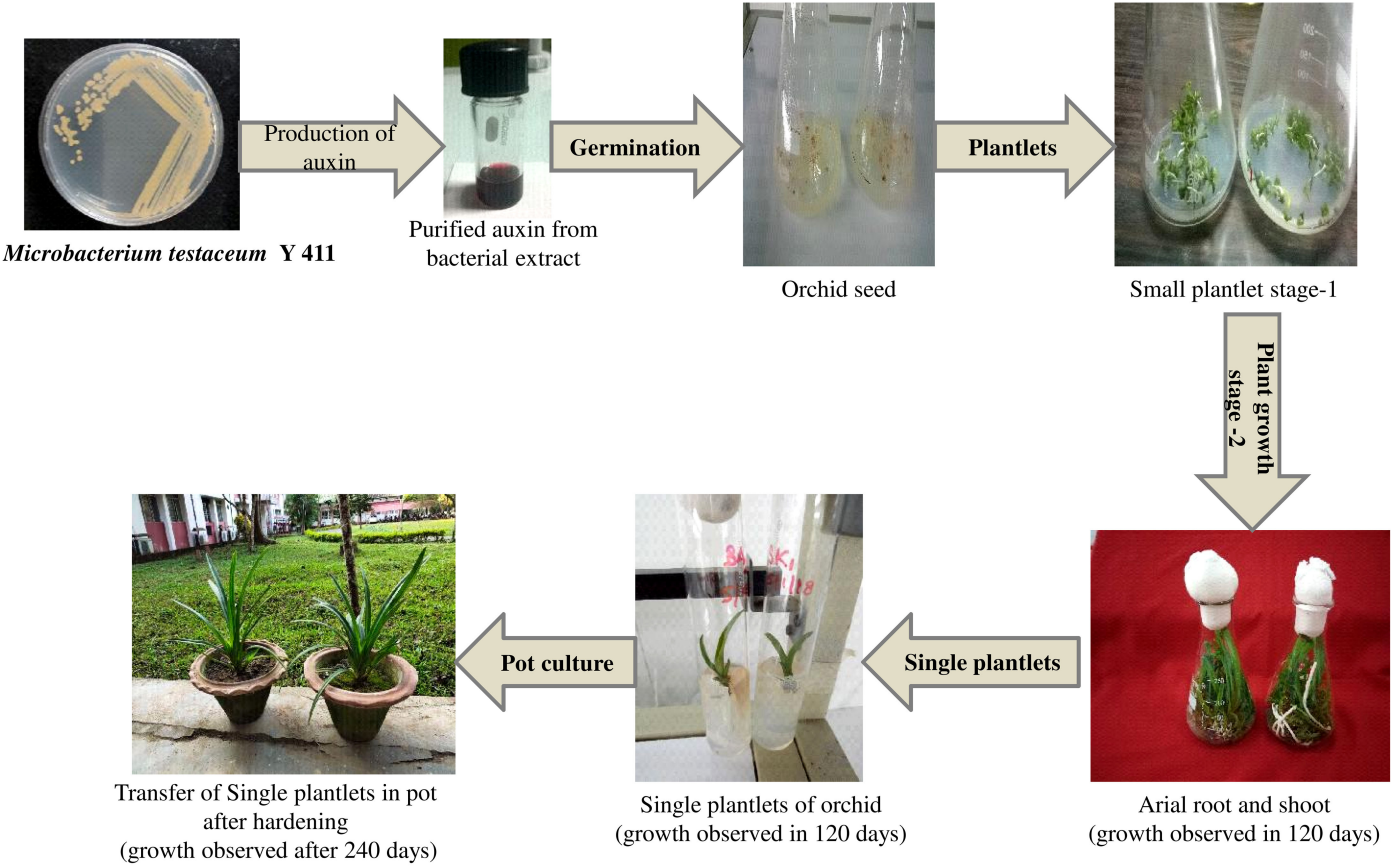
*In-vitro* propagation of the orchid (*Cymbidium aloifolium*) using purified extracted auxin from *Microbacterium testaceum* Y411.

Orchid seed germination and tissue development were investigated using pure form of bacterial auxin extracted from *Microbacterium testaceum* Y411. A combination of auxin and MS media was used to observe seed germination. In this study within 45 days, the combination of MS medium, 6-benzyl amino purine (BAP) (1 mg/L), and bacterial auxin (1 mg/L) produced the maximum seed germination (**Table 2** and **Figure 8**). Using this medium combination, the number of roots (4 Nos.), root length (4 cm), and shoot length (6.5 cm) were found to be higher. On the other hand, in the control (synthetic) media combination of MS+BAP at 1 mg/L and 1 mg/L of NAA seed germination was observed in 55 days. Plant growth metrics were revealed to be less affected by MS media with 2 mg/L of bacterium auxin and synthetic auxin (NAA) (**Table 2**). It was revealed that the culture medium with bacterial auxin had a significant effect (p<0.05) on seed germination, callus formation, root length, shoot length, and survival period **(** **Table 2** **;** **Figure 8** **).**

**Table 2:** The biological effect of bacterial auxin on orchid (*Cymbidium aloifolium*) seed germination and root induction.

Sl No	Medium	BAP (mg/L)	Bacterial auxin (mg/L)	NAA	Seed germination (%)	Time is taken for initiation of germination (days)	Callus formation (PLBS) days	Root		Shoot length (cm)	Survival rate (%)
								Root number	Root length (cm)		
1	MS medium	1.0	0.5	–	85	62	73	3	3.5	6.1	92
2	MS medium	1.0	1.0	–	90	45	57	4	4.0	6.5	97
3	MS medium	1.0	1.5	–	86	61	73	3	3.2	6.2	92
4	MS medium	1.0	2.0	–	80	67	79	2	1.6	5.8	89
5	MS medium	1.0	–	0.5	82	65	78	3	2.8	5.2	92
6	MS medium	1.0	–	1.0	87	55	68	3	3.3	5.6	93
7	MS medium	1.0	–	1.5	85	68	79	2	3.1	5.5	90
8	MS medium	1.0	–	2.0	80	70	81	2	2.7	5.1	88

*MS, medium-Murashige and Skoog medium.

## Discussion

The orchid root-associated microbial community is determined by its species or root type, where the variation may be attributed to the composition of root exudates (Kaur and Sharma, 2021). Plant exudates also provide tryptophan, the primary precursor in microbial IAA production (Kravchenko et al., 2004; Raut et al., 2017). The present investigation revealed that endophytic bacteria associated with the aerial roots of *R. retusa* capable to produce auxin (IAA). The bacterial strains were identified as *Microbacterium testaceum* based on a biochemical test and 16S rRNA gene sequence analysis. The bacterial strain was found to be a highly efficient auxin producer. The strain significantly boosted orchid growth and development through various growth-promoting properties. The earlier studies also indicated that orchid-associated bacterial species *Bacillus, Enterobacter, Pseudomonas, Stenotrphomonas*, and *Microbacterium* are used to produce auxin (Tsavkelova et al., 2007; Galdiano et al., 2011; Jakubska-Busse et al., 2021). Several reports on auxin synthesis by bacteria and their application indicate that auxins are involved in plant growth and development (Shah et al., 2021). Blinkov et al. (2014) and Chandra et al. (2018) have studied auxin’s role in the overall growth of plants and the productivity of crops by stimulating roots and shoots.

Most IAA-producing bacterial species reported as *Pseudomonas, Serratia, Stenotrophomonas, Rhizobium, Enterobacter*, etc., belong to the gram-negative class (Panigrahi et al., 2020). Except for a few species like *Microbacterium, Bacillus*, and *Streptomyces*, are gram-positive and have a high IAA production ability (Tsavkelova et al., 2007; Ek-Ramos et al., 2019). As compared to earlier reports the *Microbacterium testaceum* Y411 has produce a maximum auxin (170 µg/mL). The bacterial supernatant was used in the petri dish seed germination assay. Therefore, it is found that IAA acts as an inducer and plant growth stimulator. The plant-associated bacteria could gain an edge during plant colonization and future plant-microbe interactions. Additionally, it is evident that orchid-associated bacteria influence the symbiotic germination of mung and orchid seeds favorably (Hassani et al., 2018; Harman et al., 2021).

Temperature is an important factor affecting the enzyme activity involved in IAA production (Duca et al., 2014; Duca and Glick, 2020). The maximum production of IAA was achieved at 30 °C by *Microbacterium testaceum* Y411. Most of the bacterial species have shown better metabolism at pH 7, and several reports on IAA synthesis by bacterial species at neutral pH (Harikrishnan et al., 2014; Fu et al., 2015). It was also observed that if the incubation time was increased after 48 h, there was no substantial change in the production of IAA (Gaimster and Summers, 2015). The influence of different doses of tryptophan (250-2000 µg/mL) on the production of IAA was examined in this study. The maximum IAA production was obtained at 1000 µg/mL of tryptophan in culture media. Earlier studies reported that bacterial strains grow rapidly in the presence of 200 µg/mL to 250 µg/mL tryptophan (Naveed et al., 2015; Widawati, 2020). Similarly, *Microbacterium testaceum* Y411 growth was also recorded in the same condition. However, the production of IAA was less than 10 µg/mL. According to this study, the biomass and IAA production increased up to 1000 µg/mL with the addition of L-tryptophan in the medium. Further, increasing the concentration of tryptophan causes an inhibitory effect on metabolism resulting in lower IAA production. IAA production is majorly dependent on tryptophan (precursor of IAA), although the maximum productivity varies with the bacterial species at optimum conditions (Farahat et al., 2020; Aallam et al., 2021). The *R_f_* values of the standard and extracted auxin samples were virtually identical, i.e., about 0.53. This indicates IAA is present in the crude extracted medium. A comparable *R_f_* value for the microbial auxin was also reported by Myo et al. (2019). Previously reported studies suggest that only Salkowski assay is insufficient to confirm the auxin-based IAA molecule produced by bacteria species. HPLC is the more accurate and reliable method to identify auxin. Therefore, the bacterial extract containing IAA was confirmed using HPLC analysis. The FTIR spectrum of the bacterial extract revealed that the detected functional groups present in auxin. Further validation through HRMS results in terms of *m/z* of IAA from the extracted sample was matched with the commercially available standard compound and found the IAA metabolites (Revelou et al., 2019). The 4-chloroindole-3-acetic acid, an analog of IAA was detected from *Microbacterium testaceum* Y411 extract at *m/z* of 209.16 by HRMS analysis. A similar result was reported by Revelou et al. (2019) for plant IAA which found 4-chloroindole-3-acetic acid at *m/z* ratios 208.0167 and 210.0136. Indole compounds consist of an alkyl chain that is substituted with a positive ionization mode in the pyrrole ring, suffer splitting of the β bond relating to the aromatic compounds and bring about 3-methylene-3H-indol-1-ium ion rearrangement to quinolinium cation as a more stable with a calculated *m/z* 130.066 and 2-Methyl-1,3-dihydroindole-2-carboxylate (C_10_H_10_NO_2_) observed at *m/z* 176.

*In vitro* culture of orchids has emerging research area because of their significant economic value and high risk of extinction. For a variety of species, research is being done on culture media with different phytohormone combinations and concentration optimization (Philip, Robinson et al., 2017; Fazeli-Nasab et al., 2021; Hsu et al., 2021). It is now more crucial than ever to comprehend the optimal quantity of naturally occurring phytohormones needed for tissue culture development. In orchids, auxins are necessary for root and shoot growth, protocorm-like body (PLB) formation, and seed germination (Fang et al., 2022). *In vitro* propagation of orchids mediated through natural auxin; most researchers used crude bacterial supernatant rather than pure auxin supplied as a growth-stimulating hormone in the culture media. Present study used a pure extract of auxin from *Microbacterium testaceum* Y411 in the *in-vitro* orchid propagation. The well-grown orchid seeds were tiny and lightweight, lacking a primordial root and shoot axis. As seed germinates, the embryo cell divides into protocorm, a tubular/oblong structure of differentiated cells. The current study used seed germination to ascertain the role of auxin in protocorm development in orchids. In the MS medium containing 1 mg/L of BAP and 1 mg/L of purified bacterial auxin, seed germination took 45 days. The total number (4) of roots, the root length (4 cm), and the shoot length (6.5 cm) were all determined to be higher.

Similarly, compared to bacterial auxin, seed germination period were prolonged to 55 days in control synthetic media MS+BAP (1 mg/L) and NAA (1 mg/L). Similar results were also reported on PLB and callus formation using the combination of auxin (NAA) and cytokinin (BAP) (Pant and Swar, 2011; Dey et al., 2020). Thus, current study also proved that bacterial auxin supplemented with 1mg/L in tissue culture MS media may be a substitute for synthetic auxin.

The work’s major finding is the isolation of auxin-producing root-associated bacteria from *R. retusa.* The purified auxin of *Microbacterium testaceum* Y411 for *in vitro* orchid propagation demonstrated a significant role in plant growth and development (Novak et al., 2014; Kudoyarova et al., 2019). The present study suggests that plant growth-stimulating hormones produced by bacteria play an essential role in developing orchids. The production of phytohormones, particularly auxin, by orchid-associated bacteria is assumed to play a broad ecological function in plant fitness by altering plant hosts in diverse adverse environments. It’s also fascinating to learn more about orchid-residing bacteria and their significance in biological challenges like species preservation.

## Data availability statement

The datasets presented in this study can be found in online repositories. The names of the repository/repositories and accession number(s) can be found below: https://www.ncbi.nlm.nih.gov/genbank/, MZ367021.

## Author contributions

AY has performed the experiments, analyzed the data, and wrote the manuscript; KM is helped in the *in vitro*-propagation study; NK, SG, and PG helped for the analysis of the data; RKS is responsible for proper planning and execution of experiments and RS supervised and designed the experiments. All authors contributed to the article and approved the submitted version.

## Funding

The work was carried out under the project, NEIST-OLP-2035 funded by the Council of Scientific and Industrial Research (CSIR), Govt. of India, New Delhi.

## Acknowledgments

The authors are thankful to the Director, CSIR-NEIST, Jorhat for providing the necessary facilities to carry out the work. AY is thankful to University of Science and Technology, Meghalaya, India for her PhD work.

## Conflict of interest

The authors declare that the research was conducted in the absence of any commercial or financial relationships that could be construed as a potential conflict of interest.

## Publisher’s note

All claims expressed in this article are solely those of the authors and do not necessarily represent those of their affiliated organizations, or those of the publisher, the editors and the reviewers. Any product that may be evaluated in this article, or claim that may be made by its manufacturer, is not guaranteed or endorsed by the publisher.
